# Diagnostic utility of audiologic parameters in sudden hearing loss: a multivariable logistic regression study

**DOI:** 10.1007/s00405-026-10336-3

**Published:** 2026-06-01

**Authors:** Omer Faruk Suloglu, Mustafa Yuksel, Ayca Ciprut

**Affiliations:** 1 Present Address: Istanbul Medeniyet University, Faculty of Health Sciences, Department of Audiology, Istanbul, Türkiye; 2 Marmara University, Institute of Health Sciences, Department of Otorhinolaryngology, Audiology and Speech Disorders PhD Program, Istanbul, Türkiye; 3 Present Address: Ankara Medipol University, Faculty of Health Sciences, Department of Audiology, Ankara, Türkiye; 4 Present Address: Marmara University, Faculty of Medicine, Department of Audiology, Istanbul, Türkiye

**Keywords:** Sudden hearing loss, Sensorineural hearing loss, Logistic models, ROC curve, Diagnosis, Signs and symptoms

## Abstract

**Purpose:**

Sudden hearing loss (SHL) complaints constitute a substantial proportion of admissions to audiology clinics; however, data on individuals presenting with suspected SHL prior to diagnosis remain limited. This study aimed to describe the audiologic findings and symptoms of adult patients referred to our clinic with suspected SHL.

**Methods:**

Medical records of patients referred with suspected SHL between January 2018 and December 2019 were retrospectively reviewed. A total of 106 participants (108 ears) were included. Participants were classified into four groups based on audiologic findings, and intergroup comparisons were conducted. Logistic regression analysis was used to identify variables most strongly associated with SHL diagnosis.

**Results:**

The mean duration of hearing loss complaint was 8.8 days (SD = 8.8), and the mean pure-tone average (PTA) was 31.4 dB HL (SD = 19.9). Two participants (1.9%) had bilateral involvement, and 28.7% met the diagnostic criteria for SHL. The most frequently reported symptoms were tinnitus (63%) and ear fullness (42.6%). Logistic regression analysis indicated that the overall model was statistically significant (*p* < 0.001). Of all predictors, only the speech discrimination score (SDS) was independently associated with diagnosis (*p* < 0.05).

**Conclusion:**

The logistic regression model effectively differentiated individuals with and without SHL. Nearly half of the patients presenting with suspected SHL had normal hearing (45.4%), and 14.8% demonstrated findings consistent with middle ear pathology. These results highlight the substantial clinical burden associated with suspected SHL referrals and suggest that the coexistence of multiple symptoms may complicate the diagnostic process.

## Introduction

Sudden hearing loss (SHL) is defined as a sensorineural hearing loss (SNHL) of at least 30 dB at three consecutive frequencies developing within three days [1]. SHL is often accompanied by symptoms such as ear fullness, tinnitus, and vestibular disturbances [2–7]. A major challenge in diagnosis is the variability or absence of a clearly identifiable etiology. Potential causes include autoimmune diseases, viral infections, vascular events, ototoxic agents, cochlear membrane rupture, acoustic trauma, neurological conditions, and psychogenic factors. When no causative factor is identified, the condition is classified as idiopathic SHL [2, 3, 5, 8, 9]. The reported incidence of SHL ranges from 5 to 20 per 100,000 individuals [2]. Although SHL may occur at any age, it is most frequently observed between 45 and 60 years. While no consistently significant gender difference has been demonstrated, some studies have noted a slightly higher prevalence in women [2, 10, 11]. SHL is typically unilateral, though bilateral presentations have also been reported [2, 11–13].

A thorough medical history and a detailed evaluation of the relationship between the patient’s symptoms and audiologic findings are essential in the diagnostic process. Multiple treatment options are available for SHL, including oral corticosteroids, intratympanic steroid injections, antiviral medications, diuretics, and hyperbaric oxygen therapy. The literature also reports that hearing may improve spontaneously even in the absence of treatment [1–5, 8, 9, 11, 14]. Several factors have been suggested to influence hearing recovery, including the patient’s age, the duration and degree of hearing loss, and the presence of tinnitus or vertigo [1, 2, 5, 8, 10, 15].

A substantial body of research has focused on the audiologic findings and treatment outcomes of individuals already diagnosed with SHL. However, considerably less is known about the clinical characteristics of patients who present to audiology clinics with suspected SHL before a definitive diagnosis is established. The updated clinical practice guideline by the American Academy of Otolaryngology–Head and Neck Surgery [16] emphasizes that, at first presentation, prompt audiometry is essential to differentiate sensorineural from conductive causes rather than relying solely on symptomatology, as many suspected cases ultimately do not meet the diagnostic criteria for SHL. In daily clinical practice, this results in a high volume of urgent referrals driven by nonspecific symptoms–such as tinnitus, ear fullness, and dizziness–which frequently lead to non-SHL diagnoses and contribute to substantial clinical burden. A clearer understanding of the symptom and audiologic profiles of this referral population may therefore support more efficient triage, reduce unnecessary resource use, and streamline the diagnostic pathway for individuals requiring rapid intervention. Accordingly, the present study aimed to describe the symptoms and audiologic findings of adults referred to our audiology clinic with suspected SHL and to identify the factors most strongly associated with an eventual SHL diagnosis.

## Materials and methods

### Study design and participants

This cross-sectional and observational study was conducted at Marmara University Faculty of Medicine, Department of Audiology. The symptoms and audiologic findings of patients referred to the audiology clinic with suspected SHL between January 2018 and December 2019 were retrospectively reviewed. Ethical approval for the study was obtained from the Ethics Committee of Marmara University Faculty of Medicine (date: 03/01/2020; protocol no: 09.2020.15). All procedures were performed in accordance with the Declaration of Helsinki.

The inclusion criteria for the study were being at least 18 years of age and reporting a complaint of SHL within one month prior to evaluation. Exclusion criteria were failure to meet the inclusion criteria, incomplete medical history or audiogram data, and the presence of mental, physical, or neurological conditions that prevented audiologic testing. During the study period, 136 individuals were referred to the clinic for audiologic assessment; 30 were excluded due to incomplete medical histories or audiograms. The final sample comprised 106 participants (108 ears). All audiological assessments were conducted based on the participants’ initial tests.

All individuals with suspected SHL were categorized into one of the following groups based on audiologic findings:

- SHL Group: Patients who met the diagnostic criteria for sudden sensorineural hearing loss (≥ 30 dB sensorineural loss at three consecutive frequencies) in at least one ear.
- SNHL Group: Patients with sensorineural hearing loss in at least one ear who did not meet the SHL diagnostic criteria.
- MCHL Group: Patients with mixed or conductive hearing loss (≥ 15 dB air–bone gap) in at least one ear who did not meet the SHL diagnostic criteria.
- NH Group: Patients with normal bilateral hearing based on pure-tone averages (PTA ≤ 25 dB HL).

### Data collection

Following a detailed medical history, tympanometry was performed using the AT235 immittance meter (Interacoustics, Middelfart, Denmark) with a 226 Hz probe tone. Tympanograms were evaluated according to the Jerger classification, in which Type A reflects normal middle-ear function, Type As indicate reduced compliance, Type B indicates no measurable admittance (often associated with middle-ear effusion or obstruction), and Type C reflects negative middle-ear pressure [17].

Distortion product otoacoustic emissions (DPOAE) were measured using the Ez Screen 2 OAE system (Otodynamics, United Kingdom). At frequencies of 1, 1.5, 2, 3, 4, and 6 kHz, DPOAEs were considered present if a response was obtained at a minimum of three frequencies with a signal-to-noise ratio of ≥ 6 dB.

Pure-tone and speech audiometry were conducted in a double-walled sound-attenuated test chamber compliant with Industrial Acoustic Company (IAC) standards, using an AC-440 clinical audiometer (Interacoustics, Middelfart, Denmark). The pure-tone average (PTA) was calculated by averaging thresholds at 0.5, 1, 2, and 4 kHz. Goodman’s scale was used to classify hearing as normal based on PTA values [18].

Speech audiometry included measurement of the speech recognition threshold (SRT) and the speech discrimination score (SDS). For SRT determination, the initial presentation level was set at 20 dB above the pure-tone average (PTA). The SRT was defined as the lowest intensity level at which the participant correctly repeated at least three out of five words from a trisyllabic word list. The SDS was assessed using a list of 25 monosyllabic words presented at 40 dB above the SRT (SRT + 40 dB). The SDS was calculated as the percentage of correctly repeated words.

### Statistical analyses

All statistical analyses were conducted using jamovi software (version 2.6.19; The Jamovi Project). Categorical variables are presented as counts and percentages (“n” and “%”). Pearson’s chi-square test or Fisher’s exact test was used to analyze categorical variables. The skewness and kurtosis values of continuous variables ranged from − 1.5 to + 1.5, indicating that the data met the assumption of normality [19]. Continuous variables were compared across groups using one-way analysis of variance (ANOVA). Assumptions for ANOVA were verified; Levene’s test confirmed the equality of variances (*p* > 0.05).

For logistic regression analysis, univariable and multivariable models were constructed to examine the associations between symptoms, audiologic findings, and SHL diagnosis. The SNHL, MCHL, and NH groups were combined into a single non-SHL group, and comparisons were made between this group and the SHL group. Variables with a p value < 0.10 in the univariable analyses were considered eligible for entry into the multivariable model.

The model’s predictive power and goodness-of-fit were evaluated using the McFadden’s R^2^. Multicollinearity among independent variables was evaluated using the variance inflation factor (VIF); variables with VIF values > 10 were considered indicative of multicollinearity and were therefore not included simultaneously in the multivariable model [20]. Odds ratios (ORs) and 95% confidence intervals (CIs) were estimated. A p value < 0.05 was considered statistically significant for all analyses.

## Results

A total of 106 participants (54 males and 52 females) referred to the clinic with suspected SHL were included in the study. Two participants (1.9%) reported bilateral complaints, resulting in the evaluation of 108 ears (54 right and 54 left). Participants ranged in age from 18 to 73 years, with a mean age of 43.0 years (SD = 14.7). The mean duration of hearing loss complaint was 8.8 days (SD = 8.8), and the mean PTA was 31.4 dB HL (SD = 19.9).

Of the participants, 28.7% (*n* = 31) were classified into the SHL group, 11.1% (*n* = 12) into the SNHL group, 14.8% (*n* = 16) into the MCHL group, and 45.4% (*n* = 49) into the NH group. The most frequently reported symptoms were tinnitus (63%) and ear fullness (42.6%). The characteristics of the participants, including audiologic findings and symptom distributions, are presented in Table 1.

**Table 1:** Characteristics of participants, audiologic findings, and symptom distribution

FactorsM (SD), n (%)	Groups n (%)					FET or χ2	P value
	SHL31 (28.7%)	SNHL12 (11.1%)	MCHL16 (14.8%)	NH49 (45.4%)	Total108 (100%)		
Age (years)	49.7 (14.2)	45.5 (11.2)	50.5 (17.0)	35.7 (11.4)	43 (14.7)		
Duration of hearing loss complaint (days)	10.2 (10.0)	12.5 (9.9)	9.1 (9.0)	7.0 (7.3)	8.8 (8.8)		
PTA	49.7 (14.9)	32.9 (3.9)	48.7 (13.7)	13.8 (7.0)	31.4 (19.9)		
SRT	47.7 (19.7)	27.5 (9.9)	43.1 (12.2)	13.9 (7.0)	29.4 (19.9)		
SDS	73.1 (21.3)	94 (6.5)	86.8 (12.2)	95.5 (9.8)	87.6 (17.0)		
Gender							
Male	14 (13%)	9 (8.3%)	7 (6.5%)	23 (21.3%)	53 (49.1%)	3.69^▲^	0.297
Female	17 (15.7%)	3 (2.8%)	9 (8.3%)	26 (24.1%)	55 (50.9%)		
Affected Ear							
Right	20 (18.5%)	5 (4.6%)	8 (7.4%)	21 (19.4%)	54 (50%)	3.95^▲^	0.267
Left	11 (10.2%)	7 (6.5%)	8 (7.4%)	28 (25.9%)	54 (50%)		
DPOAE							
Yes	5 (4.6%)	4 (3.7%)	1 (0.9%)	49 (45.4%)	59 (54.6%)	76.54^■^	< .001*
No	26 (24.1%)	8 (7.4%)	15 (13.9%)	0 (0%)	49 (45.4%)		
Tympanogram						100.1^■^	< .001*
A	31 (28.7%)	12 (11.1%)	1 (0.9%)	49 (45.4%)	93 (86.1%)		
As	0 (0%)	0 (0%)	5 (4.6%)	0 (0%)	5 (4.6%)		
B	0 (0%)	0 (0%)	8 (7.4%)	0 (0%)	8 (7.4%)		
C	0 (0%)	0 (0%)	2 (1.9%)	0 (0%)	2 (1.9%)		
Head Trauma							
Yes	2 (1.9%)	2 (1.9%)	3 (2.8%)	6 (5.6%)	13 (12%)	1.84^■^	0.545
No	29 (26.9%)	10 (9.3%)	13 (12%)	43 (39.8%)	95 (88%)		
Noise exposure							
Yes	5 (4.6%)	7 (6.5%)	3 (2.8%)	11 (10.2%)	26 (24.1%)	9.09^▲^	0.028*
No	26 (24.1%)	5 (4.6%)	13 (12%)	38 (35.2%)	82 (75.9%)		
Drag use							
Yes	5 (4.6%)	4 (3.7%)	4 (3.7%)	11 (10.2%)	24 (22.2%)	1.60^■^	0.605
No	26 (24.1%)	8 (7.4%)	12 (11.1%)	38 (35.2%)	84 (77.8%)		
Diabetes							
Yes	1 (0.9%)	1 (0.9%)	1 (0.9%)	1 (0.9%)	4 (3.7%)	1.41^■^	0.478
No	30 (27.8%)	11 (10.2%)	15 (13.9%)	48 (44.4%)	104 (96.3%)		
High blood pressure							
Yes	3 (2.8%)	0 (0%)	1 (0.9%)	1 (0.9%)	5 (4.6%)	3.21^■^	0.363
No	28 (25.9%)	12 (11.1%)	15 (13.9%)	48 (44.4%)	103 (95.4%)		
Tinnitus							
Yes	18 (16.7%)	7 (6.5%)	7 (6.5%)	36 (33.3%)	68 (63%)	5.28^▲^	0.152
No	13 (12%)	5 (4.6%)	9 (8.3%)	13 (12%)	40 (37%)		
Ear fullness							
Yes	12 (11.1%)	4 (3.7%)	3 (2.8%)	27 (25%)	46 (42.6%)	7.47^■^	0.061
No	19 (17.6%)	8 (7.4%)	13 (12%)	22 (20.4%)	62 (57.4%)		
Vestibular symptom							
Yes	10 (9.3%)	2 (1.9%)	2 (1.9%)	10 (9.3%)	24 (22.2%)	2.99^■^	0.468
No	21 (19.4%)	10 (9.3%)	14 (13%)	39 (36.1%)	84 (77.8%)		
SHL history							
Yes	6 (5.6%)	1 (0.9%)	1 (0.9%)	3 (2.8%)	11 (10.2%)	4.05^■^	0.277
No	25 (23.1%)	11 (10.2%)	15 (13.9%)	46 (42.6%)	97 (89.8%)		

*SHL*sudden hearing loss;*SNHL*sensorineural hearing loss; *MCHL*mixed/conductive hearing loss; *M*mean; *SD*standard deviation; *χ*2 Pearson’s chi-square test; *FET* Fisher’s exact test; *: *p* < 0.05; ^▲^: Pearson’s chi-square value; ^■^: Fisher’s Exact Test value

Group differences in continuous variables (age, duration of hearing loss complaint, PTA, SRT, and SDS) were examined using one-way ANOVA. A statistically significant difference was observed in age (F(3,104) = 9.69, *p* < 0.001, η²*p* = 0.22). Tukey post hoc analysis revealed that the SHL and MCHL groups were significantly older than the NH group (both *p* < 0.001; Cohens’ d = 1.07 and 1.13, respectively). No other pairwise differences in age reached statistical significance (all *p* > 0.05). Duration of hearing loss complaint did not differ significantly among the groups (F(3,104) = 1.71, *p* = 0.169, η²*p* = 0.05).

PTA values differed significantly between groups (F(3,104) = 87.31, *p* < 0.001, η²*p* = 0.72). Post hoc analysis showed that the NH group had significantly lower PTA values than the SHL, SNHL and MCHL groups (all *p* < 0.001; Cohens’ d = 3.33, 1.78, and 3.24, respectively). The SNHL group also had significantly lower PTA values than both the SHL and MCHL groups (both *p* < 0.001; Cohens’ d = 1.56, and 1.47, respectively).

A significant group difference was also found for SRT (F(3,104) = 50.76, *p* < 0.001, η²*p* = 0.59). The NH group had significantly lower SRT values than the SHL (*p* < 0.001, Cohens’ d = 2.63), SNHL (*p* = 0.008, Cohens’ d = 1.06) and MCHL groups (*p* < 0.001, Cohens’ d = 2.27). Additionally, the SNHL group demonstrated significantly lower SRT values than the SHL and MCHL groups (*p* < 0.001, Cohen’s d = 1.57; *p* = 0.010, Cohen’s d = 1.21, respectively).

SDS values also differed significantly among groups (F(3,104) = 16.69, *p* < 0.001, η²*p* = 0.32). Tukey post hoc comparisons showed that the SHL group had significantly lower SDS values than the SNHL (*p* < 0.001, Cohen’s d = 1.48), MCHL (*p* = 0.012, Cohen’s d = 0.96), and NH groups (*p* < 0.001, Cohen’s d = 1.58).

For categorical variables, Fisher’s exact test indicated statistically significant associations among the groups for both DPOAE outcomes (Cramer’s V = 0.84, *p* < 0.001) and tympanogram types (Cramer’s V = 0.56, *p* < 0.001), both representing large effect sizes. Pearson’s chi-square test showed a significant relationship in noise-exposure history across groups, reflecting a moderate effect size (χ²(3) = 9.09, *p* = 0.028, Cramer’s V = 0.29). No significant relationships were found for the remaining symptoms (see Table 1).

The SNHL, MCHL, and NH groups were combined into a non-SHL group for logistic regression analysis. Prior to performing the logistic regression analysis, several diagnostic tests were conducted to ensure the model assumptions were met. Multicollinearity was assessed using VIF, with all values remaining below the threshold of 10 ranging from 1.06 to 7.20, indicating no significant overlap between independent variables. Additionally, the model’s predictive power and goodness-of-fit were evaluated using the McFadden’s R^2^. The resulting value of 0.381 indicates a robust model fit. The overall model was statistically significant (χ²(7, *N* = 108) = 49.38, *p* < 0.001). Univariable analyses identified age, PTA, SRT, SDS, and DPOAE as significant predictors (all *p* < 0.001 except age; *p* = 0.003). Variables with *p* < 0.10 in the univariable analysis, including affected ear and history of SHL (*p* = 0.058 and *p* = 0.056, respectively), were included in the multivariable model. After adjustment for confounding variables, only SDS remained independently associated with SHL diagnosis (*p* = 0.043, OR: 0.96; see Table 2).

**Table 2:** Logistic regression results

Variables	Unadjusted results (Univariable analysis)			Adjusted results (Multivariable analysis)		
	OR	95% CI lower (upper)	*P* value	OR	95% CI lower (upper)	*P* value
Age (year)	1.05	1.02 (1.08)	0.003*	0.98	0.94 (1.03)	0.425
Duration of hearing loss complaint (day)	1.02	0.98 (1.07)	0.314			
PTA	1.09	1.05 (1.12)	< 0.001*	1.04	0.96 (1.13)	0.308
SRT	1.08	1.05 (1.12)	< 0.001*	1.01	0.93 (1.09)	0.882
SDS	0.93	0.90 (0.96)	< 0.001*	0.96	0.92 (1.00)	0.043*
Gender (ref: Male) Female	1.25	0.54 (2.88)	0.606	-	-	-
Affected Ear (ref: Left) Right	2.30	0.97 (5.45)	0.058**	2.15	0.67 (6.88)	0.198
DPOAE (ref: Yes) No	12.21	4.17 (35.75)	< 0.001*	2.17	0.39 (12.13)	0.378
Head Trauma (ref: Yes) No	2.42	0.50 (11.60)	0.270	-	-	-
Noise exposure (ref: Yes) No	1.95	0.66 (5.75)	0.226	-	-	-
Use of drugs (ref: Yes) No	1.70	0.57 (5.06)	0.337	-	-	-
Diabetes (ref: Yes) No	1.22	0.12 (12.16)	0.868	-	-	-
HBP (ref: No) Yes	4.02	0.64 (25.33)	0.139	-	-	-
Tinnitus (ref: Yes) No	1.34	0.57 (3.14)	0.504	-	-	-
Ear fullness (ref: Yes) No	1.25	0.53 (2.93)	0.605	-	-	-
Vertigo (ref: No) Yes	2.14	0.83 (5.54)	0.116	-	-	-
SHL history (ref: No) Yes	3.46	0.97 (12.32)	0.056**	2.90	0.52 (16.23)	0.225

*HBP* High Blood Pressure; *SHL* Sudden Hearing Loss; *OR* Odds Ratio; *CI* Confidence Interval; ref: reference; *: *p* < 0.05; **: *p* < 0.1

**Fig. 1:**
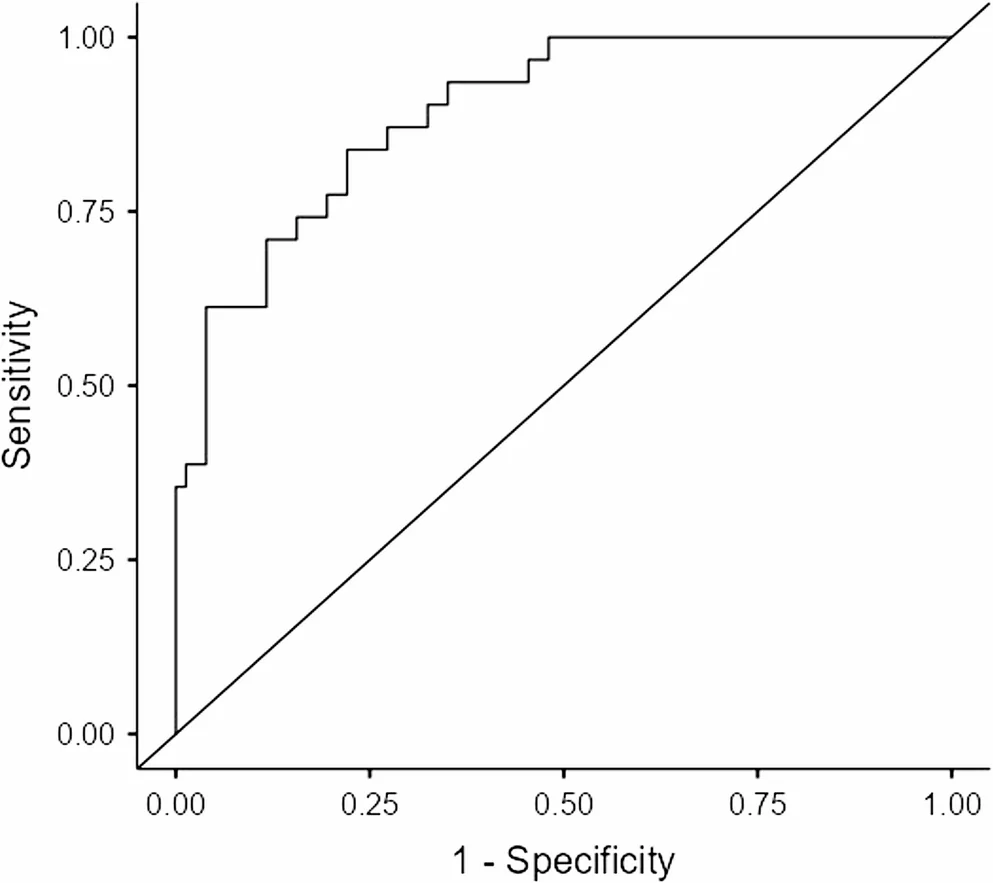
ROC curve

The logistic regression model demonstrated 82.4% accuracy, with 61.3% sensitivity and 90.9% specificity. The positive predictive value (PPV) was 73.1%, and the negative predictive value (NPV) was 85.4%. The positive likelihood ratio (LR+) was 6.7, and the negative likelihood ratio (LR–) was 0.43. The area under the receiver operating characteristic curve (AUC) was 0.89, indicating excellent discriminative ability. The receiver operating characteristic (ROC) curve is presented in Fig. 1, and diagnostic performance indices are summarized in Table 3.

**Table 3:** Diagnostic performance indices

	Observed Yes (31)	Observed No (77)	Total
Predicted Yes	TP = 19	FP = 7	26
Predicted No	FN = 12	TN = 70	82
Total	31	77	108

*TP* true positive; *FP* false positive; *TN* true negative; *FN* false negative

## Discussion

Individuals presenting with SHL complaints constitute a substantial proportion of patients seeking urgent audiologic evaluation. In the present study, we evaluated the symptoms and audiologic profiles of adults referred with suspected SHL prior to definitive diagnosis. A key finding was that the majority of these referrals ultimately did not result in an SHL diagnosis: 71.3% of patients were classified into non-SHL groups, and nearly half (45.4%) demonstrated normal hearing. These results suggest that while SHL is perceived as an alarming condition requiring immediate care, most patients who seek urgent evaluation for SHL-like symptoms do not meet diagnostic criteria. This pattern underscores a significant referral burden on audiology clinics and highlights the importance of effective differentiation at the point of initial presentation.

### Symptom presentation and implications for differential diagnosis

Ear fullness (55.1%) and tinnitus (73.5%) were most frequently reported in the NH group, indicating that these complaints–although common in SHL–are not specific indicators of SHL and may prompt unnecessary emergency referral. Spontaneous recovery rates for SHL range from 32% to 65% [21, 22], raising the possibility that some NH cases may represent individuals who experienced recovery before being assessed. A similar mechanism may apply to the MCHL group (14.8%), in which symptoms such as ear fullness and tinnitus may have been initially misinterpreted as signs of SHL. For these patients, detailed middle-ear evaluation–including otoscopy and tuning fork tests–is necessary to rule out conductive or mixed pathologies, consistent with the recommendations [16, 22]. In addition, the removal of impacted cerumen is advised before SHL diagnosis when indicated [23]. Within the SNHL group (11.1%), the rapid progression of hearing loss may have led patients to assume SHL, prompting clinical consultation; this may be associated with the presence of noise-exposure history in more than half of these participants (58.3%).

The prevalence of tinnitus, ear fullness, and vertigo among confirmed SHL cases in our study–58%, 39%, and 32%, respectively–is broadly aligned with patterns observed in ENT and audiology clinics. However, because these symptoms are also widely reported in non-SHL pathologies, they do not provide diagnostic specificity. This observation is supported by the findings of the multivariable logistic regression model, in which tinnitus, ear fullness, vestibular symptoms, and other clinical complaints did not independently predict SHL, further reinforcing the idiopathic nature of SHL described in the literature [7, 11, 22].

### Demographic and clinical timeline characteristics

Intervention within the first three days is considered critical in SHL, although treatment may still offer benefit when diagnosis is delayed [1, 5, 8, 10, 11, 15]. Consistent with prior studies [1, 2, 4, 11, 14, 24], we included participants who presented within one month of symptom onset. The mean duration of hearing loss complaint was 8.8 days (SD = 8.8), overlapping with the spontaneous recovery period associated with seasonal allergies, upper respiratory tract infections, and idiopathic SHL [24]. This timeline may help explain both the modest proportion of confirmed SHL cases (28.7%) and the lack of significant group differences in hearing loss complaint duration (*p* = 0.169, η²*p* = 0.05).

Our findings also mirrored prior reports indicating that SHL is most frequently observed in individuals in their 40s [2, 4, 7, 10, 11, 24], although it can occur much later in life [1]. The NH group had the lowest mean age, which may have contributed to the significant age difference across groups (*p* < 0.001, η²*p* = 0.22). Gender distribution did not differ significantly across groups (*p* = 0.297), although the SHL group included a higher proportion of women, consistent with previous literature [2, 4, 6, 10, 11, 15]. While the right and left ears were equally represented across the total sample, right-ear involvement was more common in the SHL group (64.5%), aligning with some previous studies [4, 15]. However, conflicting reports exist regarding laterality [2, 5, 24], suggesting that the etiology likely varies across individuals.

### Audiologic and otologic characteristics

Audiologic measures demonstrated clear differentiation across patient groups. The SHL and MCHL groups exhibited the highest and most similar PTA and SRT values, which likely explains the lack of a statistically significant difference between these two groups, despite both being significantly elevated compared with the NH and SNHL groups (all *p* < 0.001). Mean PTA and SRT values in our SHL group were lower than those typically reported in the literature [4, 5, 24], which may reflect differences in sample characteristics or sample size. SDS findings showed a similar pattern: SDS was lowest in the SHL group, consistent with the literature, though higher in absolute magnitude compared with prior studies–potentially due to lower PTA and SRT values in the present sample.

Robust otologic distinctions were also observed across groups. DPOAE responses were preserved in all NH participants, while only 16.1% of patients in the SHL group demonstrated emissions, consistent with previous reports that SHL is associated with outer hair cell dysfunction [25, 26]. Tympanometric differences reflected the clustering of Type As, B, and C tympanograms in the MCHL group, and the higher rate of noise exposure in the SNHL group (58.3%) may help explain their clinical presentation. Bilateral involvement was rare (1.9%), consistent with previously reported rates between 1.7% and 2% [2, 5, 6, 11, 12].

### Predictors of SHL diagnosis: logistic regression analysis

In circumstances where multiple factors may influence outcomes, multivariable analyses that allow adjustment for confounding variables become essential. Logistic regression analysis constitutes a multivariable analysis model that has the capacity to examine the relationship between several factors and disease [27]. Although PTA, SRT, and SDS were significant predictors in univariable analyses, only SDS remained statistically significant in the multivariable model (*p* = 0.043, OR: 0.96). This finding suggests that PTA and SRT may function largely as confounding variables, whereas SDS captures diagnostic information that is not redundant with other audiologic measures. The diagnostic relevance of SDS is also supported by its consistently lower values in the SHL group compared with all other groups. Although age and DPOAE were significant predictors in the univariable model, they did not retain significance in the multivariable analysis, likely due to intercorrelations among variables. The high odds ratio observed for the history of SHL may indicate potential clinical relevance; however, the wide confidence interval and non-significant p value require cautious interpretation. Future large-scale, prospective studies may clarify whether this factor contributes meaningfully to diagnostic decision-making.

### Study limitations and strengths

This single-center, retrospective design may be considered a limitation when compared with studies incorporating larger and more diverse samples. Nevertheless, the 2018–2019 time frame was intentionally selected because the onset of the Coronavirus Disease (COVID-19) pandemic in 2020 was expected to influence the reliability of symptom reporting and clinical referral patterns.

Another limitation is that, although patients were clinically evaluated by specialists and neurological disorders were ruled out as a result of routine clinical evaluations, imaging findings (e.g., MRI) could not be systematically obtained due to the study’s retrospective design and were, therefore, not included in the analysis. Additionally, future logistic regression studies could include extended high-frequency analyses to better characterize audiometric patterns in SHL. High-frequency thresholds may provide additional insight into the audiometric configuration of hearing loss.

A key strength of the present study is its unique focus on individuals presenting with suspected SHL prior to definitive diagnosis–an aspect that has received limited attention in the literature. Existing studies predominantly evaluate treatment outcomes among patients already diagnosed with SHL, whereas examining symptom profiles during the diagnostic phase is equally critical for clinical decision-making and prognosis. Furthermore, the diagnostic-oriented research design adopted here, particularly the use of multivariable logistic regression analysis, provides insight into the independent contribution of clinical and audiologic factors, thereby adding meaningful value to the current body of knowledge.

## Conclusion

This study demonstrated that most individuals referred to audiology clinics with suspected SHL do not ultimately meet the diagnostic criteria for SHL, with nearly half presenting normal hearing thresholds. These findings indicate that SHL-like symptoms frequently prompt urgent care seeking despite the absence of SHL, underscoring the need for efficient diagnostic differentiation at the point of first presentation.

Among all clinical and audiologic variables examined, the SDS emerged as the only independent predictor of SHL in the multivariable logistic regression analysis. This suggests that SDS may provide unique diagnostic value beyond that captured by pure-tone thresholds and SRT.

Collectively, these results highlight the importance of a structured evaluation protocol for patients presenting with SHL-like complaints–prioritizing careful analysis of audiologic findings, judicious interpretation of symptom reports, and timely differentiation between sensorineural, conductive/mixed, and non-pathologic cases. Such an approach has the potential to reduce unnecessary clinical burden, streamline referral pathways, and accelerate the initiation of treatment for those who require urgent intervention.

## Data Availability

The data are not publicly available due to privacy or ethical restrictions.
